# Ghrelin therapy improves lung and cardiovascular function in experimental emphysema

**DOI:** 10.1186/s12931-017-0668-9

**Published:** 2017-11-03

**Authors:** Nazareth de Novaes Rocha, Milena Vasconcellos de Oliveira, Cássia Lisboa Braga, Gabriela Guimarães, Lígia de Albuquerque Maia, Gisele de Araújo Padilha, Johnatas Dutra Silva, Christina Maeda Takiya, Vera Luiza Capelozzi, Pedro Leme Silva, Patricia Rieken Macedo Rocco

**Affiliations:** 1 Laboratory of Pulmonary Investigation, Carlos Chagas Filho Institute of Biophysics, Federal University of Rio de Janeiro, Centro de Ciências da Saúde, Avenida Carlos Chagas Filho, s/n, Bloco G-014, Ilha do Fundão, Rio de Janeiro, RJ 21941-902 Brazil; 2 National Institute of Science and Technology for Regenerative Medicine, Rio de Janeiro, Brazil; 3 Department of Physiology and Pharmacology, Biomedical Institute, Fluminense Federal University, Rio de Janeiro, Brazil; 4 Laboratory of Immunopathology, Carlos Chagas Filho Institute of Biophysics, Federal University of Rio de Janeiro, Rio de Janeiro, Brazil; 5 Department of Pathology, Faculty of Medicine, University of São Paulo, São Paulo, Brazil

**Keywords:** Experimental emphysema, Ghrelin therapy, Lung inflammation, Lung remodelling, Lung function, Cardiac function

## Abstract

**Background:**

Emphysema is a progressive disease characterized by irreversible airspace enlargement followed by a decline in lung function. It also causes extrapulmonary effects, such as loss of body mass and *cor pulmonale*, which are associated with shorter survival and worse clinical outcomes. Ghrelin, a growth-hormone secretagogue, stimulates muscle anabolism, has anti-inflammatory effects, promotes vasodilation, and improves cardiac performance. Therefore, we hypothesized that ghrelin might reduce lung inflammation and remodelling as well as improve lung mechanics and cardiac function in experimental emphysema.

**Methods:**

Forty female C57BL/6 mice were randomly assigned into two main groups: control (C) and emphysema (ELA). In the ELA group (n=20), animals received four intratracheal instillations of pancreatic porcine elastase (PPE) at 1-week intervals. C animals (n=20) received saline alone (50 μL) using the same protocol. Two weeks after the last instillation of saline or PPE, C and ELA animals received ghrelin or saline (n=10/group) intraperitoneally (i.p.) daily, during 3 weeks. Dual-energy X-ray absorptiometry (DEXA), echocardiography, lung mechanics, histology, and molecular biology were analysed.

**Results:**

In elastase-induced emphysema, ghrelin treatment decreased alveolar hyperinflation and mean linear intercept, neutrophil infiltration, and collagen fibre content in the alveolar septa and pulmonary vessel wall; increased elastic fibre content; reduced M1-macrophage populations and increased M2 polarization; decreased levels of keratinocyte-derived chemokine (KC, a mouse analogue of interleukin-8), tumour necrosis factor-α, and transforming growth factor-β, but increased interleukin-10 in lung tissue; augmented static lung elastance; reduced arterial pulmonary hypertension and right ventricular hypertrophy on echocardiography; and increased lean mass.

**Conclusion:**

In the elastase-induced emphysema model used herein, ghrelin not only reduced lung damage but also improved cardiac function and increased lean mass. These findings should prompt further studies to evaluate ghrelin as a potential therapy for emphysema.

**Electronic supplementary material:**

The online version of this article (10.1186/s12931-017-0668-9) contains supplementary material, which is available to authorized users.

## Background

Emphysema is a progressive disease characterized by irreversible airspace enlargement, followed by a decline in lung function [1]. The imbalance between elastase and anti-elastase activity [2], rupture of alveolar walls [3], and inflammation in the lung parenchyma [4] are some of the hallmarks of this disease. In addition to the well-known impact of emphysema on the lungs, extrapulmonary effects have also been described, such as pulmonary arterial hypertension, *cor pulmonale,* and changes in right ventricular structure and function [5]; skeletal muscle wasting [6]; and body weight loss [7]. These systemic manifestations are associated with increased risk of exacerbation and decreased survival [8].

The current therapeutic approach for emphysema is mainly focused on the use of bronchodilators, anti-inflammatory agents, and antibiotics [1]. To date, there has been no effective therapy able to modify the long-term decline in lung function. Therefore, a new pharmacological therapy able to reduce inflammation and remodelling, as well as mitigate the extrapulmonary effects associated with emphysema, might represent a potential disease-modifying strategy.

In this context, ghrelin, an endogenous ligand for the growth hormone secretagogue receptor (GHS-R) produced primarily by ghrelinergic cells in the stomach [9, 10], has direct effects on muscle anabolism and appetite stimulation [11, 12]. When administered exogenously, ghrelin has anti-inflammatory and anti-apoptotic effects [10], suppresses sympathetic activity [13], and induces vasodilation, followed by improvement of cardiac performance [14, 15]. Clinical studies have demonstrated that exogenous ghrelin administration is safe and can improve muscle strength, but its effects on cardiorespiratory parameters are unclear [16–18], which may be explained by small sample sizes [16, 19].

Recently, in a cigarette smoke-exposed rat model of emphysema, subcutaneous ghrelin administration was shown to increase body weight and improve respiratory function [20]. However, ghrelin was administered only during emphysema induction and not after emphysema was established, thus limiting the translational aspect of the study. Additionally, the role of ghrelin in cardiovascular impairment, which is associated with worse prognosis [16], was not evaluated.

We hypothesised that, in a well-established model of elastase-induced severe emphysema, with clearly observable lung damage and *cor pulmonale*, therapy with ghrelin might reduce pulmonary inflammation and remodelling, as well as improve lung mechanics and cardiac function.

## Methods

### Ethics statement

This study was approved by the Animal Ethics Committee of the Health Sciences Centre (CEUA: 183/13), Federal University of Rio de Janeiro. All animals received humane care in compliance with the “Principles of Laboratory Animal Care” formulated by the National Society for Medical Research and the U.S. National Research Council “Guide for the Care and Use of Laboratory Animals”. The present study followed the ARRIVE guidelines for reporting of animal research [21]. Animals were housed in standard laboratory cages (12-h light/dark cycles, temperature 23 ± 1°C), each one containing groups of 3 animals. Mice had access to food and water *ad libitum*.

### Animal preparation and experimental protocol

**Fig. 1:**
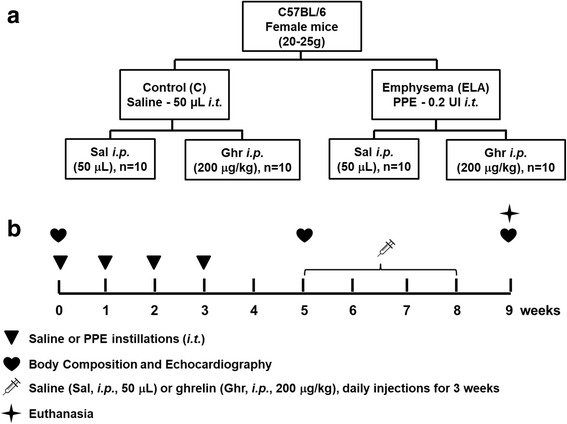
Schematic flow chart (**a**) and timeline of study design (**b**). C group: intratracheal instillation of 50 μL of saline, ELA: intratracheal instillation of 0.2 UI of pancreatic porcine elastase (PPE) once a week during 4 weeks, Sal: i.p. injection of saline (50 μL), Ghr: i.p. injection of ghrelin (200 μg kg^-1^ per day)

Forty females C57BL/6 mice (weight 20–25 g, age 2 months) were randomly assigned using closed sealed envelopes into two main groups: control (C) and emphysema (ELA). In the ELA group (n=20), animals received four intratracheal instillations of pancreatic porcine elastase (PPE) (E1250, Sigma Chemical Co., St Louis, MO, USA; 0.2 IU in 50 μL saline) at 1-week intervals (total dose, 0.8 IU PPE). C animals (n=20) received saline alone (0.9% NaCl, 50 μL) using the same protocol (Fig. 1a). For all intratracheal instillations, which occurred in the laboratory between 9 a.m. and 12 a.m., mice were anaesthetised with inhaled sevoflurane 2% (Sevorane®, Cristália, Itapira, SP, Brazil). A midline cervical incision (1 cm) was made to expose the trachea, and saline or PPE were instilled using a bent 27-gauge tuberculin needle. The cervical incision was closed with 5-0 silk suture, and the mice returned to their cages. All animals received tramadol [(50 mg kg^-1^, intraperitoneally (i.p.), Tramadon®, Cristália, Itapira, SP, Brazil] after the surgical procedure. Two weeks after the last instillation of saline or PPE, animals were further randomized into subgroups (*n*=10/group) that received saline solution (0.9% NaCl, 50 μL) or ghrelin (SP-GHRL-5, 200 μg kg^-1^ per day in 50 μL saline) i.p., daily, during 3 weeks (Fig. 1b). Ghrelin [acyl form, Ser (n-Octanolyl)] was purchased from Innovagen (Lund, Sweden) and administered in accordance with the manufacturer’s instructions. All protocols were performed in blind fashion.

### Body composition analysis

Body composition (total, fat, and lean masses) was evaluated by dual-energy X-ray absorptiometry (DEXA, Hologic QDR 4500, Hologic, Bedford, MA, USA) [22]. Prior to scanning, mice were sedated by intraperitoneal administration of diazepam [(10 mg kg^-1^), Compaz®, Cristália, Itapira, SP, Brazil].

### Echocardiography

To evaluate cardiac function in response to pulmonary emphysema and/or ghrelin therapy, echocardiography was performed at three time points: before the first elastase or saline instillation; 2 weeks after the last elastase or saline instillation; and 1 week after the last administration of saline or ghrelin. For echocardiographic assessment of cardiac function, the animals were anesthetised with inhaled sevoflurane 2%, shaved over the precordial region, and examined with a Vevo 770 system (VisualSonics®, Toronto, ON, Canada) coupled to a 30-MHz transducer. Images were obtained from the parasternal, short-axis, and long-axis views. B-dimensional parasternal short axis views of both ventricles were acquired at the level of the papillary muscles to obtain left and right ventricular areas. Pulsed-wave Doppler was used to measure pulmonary artery acceleration time (PAT), pulmonary artery ejection time (PET), and their ratio (PAT/PET), an indirect index of pulmonary arterial hypertension [23]. Left ventricular stroke volume (LVSV) was calculated by the Teichholz formula. All parameters followed American Society of Echocardiography and European Association of Cardiovascular Imaging recommendations [24].

### Lung mechanics

One week after the last administration of saline or ghrelin (Fig. 1b), animals were pre-medicated with diazepam [(10 mg kg^-1^, i.p.) Compaz®, Cristália, Itapira, SP, Brazil], anaesthetised with thiopental sodium [(20 mg kg^-1^ i.p.) Thiopentax®, Cristália, Itapira, SP, Brazil], tracheotomised, paralysed with vecuronium bromide 0.005 mg kg^-1^ i.v. (Vecuron®, Cristália, Itapira, SP, Brazil), and ventilated with a constant flow ventilator (Samay VR15; Universidad de la Republica, Montevideo, Uruguay) set as follows: respiratory rate 100 breaths/min, tidal volume (V_T_) 0.2 mL, and fraction of inspired oxygen (FiO_2_) 0.21. The anterior chest wall was surgically removed and a positive end-expiratory pressure (PEEP) of 2 cmH_2_O applied. Airflow and tracheal pressure (Ptr) were measured. In an open chest preparation, Ptr reflects transpulmonary pressure (PL). Lung mechanics were analysed by the end-inflation occlusion method [25, 26]. Static lung elastance (Est,L) was determined by dividing lung elastic recoil pressure (Pel) by V_T_. Est,L was measured 10 times in each animal. All data were analysed using ANADAT software (RHT-InfoData, Inc., Montreal, Quebec, Canada). All experiments lasted less than 15 min.

### Lung histology

At the end of the experiment, laparotomy was performed immediately after determination of lung mechanics and 1000 IU of heparin (Hemofol®, Cristália, Itapira, SP, Brazil) was injected into the vena cava. The trachea was clamped at end-expiration (PEEP = 2 cmH_2_O) in all groups to avoid distortion of lung morphometry [26, 27]. Mice were killed by exsanguination following transection of the abdominal aorta and vena cava. The left lung was then removed, fixed in 3% buffered formalin, and embedded in paraffin. Slices 4 μm thick were cut and stained with haematoxylin–eosin. Lung histology analysis was performed with an integrating eyepiece with a coherent system consisting of a grid with 100 points and 50 lines of known length coupled to a conventional light microscope (Olympus BX51, Olympus Latin America Inc., Brazil). The volume fractions of the lung occupied by collapsed alveoli (alveoli with rough or plicate walls), normal pulmonary areas, or hyperinflated structures (alveolar ducts, alveolar sacs, or alveoli, all with maximal chord length in air >120 mm) were determined by the point-counting technique [28] across 10 random, non-coincident microscopic fields. Briefly, points falling on collapsed, normal pulmonary areas or hyperinflated structures were counted and divided by the total number of points in each microscopic field. Enlargement of air spaces was evaluated using mean linear intercept measurement. The number of total cells, neutrophils, and mononuclear cells, as well as the amount of pulmonary tissue, were also determined by the point-counting technique across 10 random, non-coincident microscopic fields at ×1,000 magnification. Data were reported as the fraction area of pulmonary tissue.

The amount of collagen fibres (Picrosirius polarization method) was computed in alveolar septa and pulmonary vessel walls. Elastic fibre content was computed in alveolar septa using Weigert’s resorcin–fuchsin method with oxidation. The images were generated by a microscope (Axioplan, Zeiss) connected to a digital camera (Sony Trinitron CCD, Sony, Tokyo, Japan) and fed into a computer through a frame grabber (Oculus TCX, Coreco, St Laurent, QC, Canada) for offline processing. The thresholds for collagen and elastic fibres were established after enhancement of contrast up to the point where fibres were easily identified as either birefringent (collagen) or black (elastic) bands at ×400 magnification, in Image-Pro Plus 7.1 Software (Media Cybernetics, Silver Spring, MD, USA) [29]. The areas occupied by the elastic and collagen fibres were measured by digital densitometric recognition, divided by the tissue of each studied area, and expressed as the percentage of elastic or collagen fibre in the alveolar septa or pulmonary vessel wall.

### Immunohistochemistry

Immunohistochemical analysis for alveolar macrophages was characterized using F4/80 antibody (1:50; MCA497, AbD Serotec, Raleigh, NC, USA). Immunohistochemical analysis for M1 and M2 macrophages in lung tissue was performed using inducible nitric oxide synthase rabbit anti-mouse polyclonal antibody (M1, Rb-9242, Thermo Fischer Scientific, MA, USA) and arginase-1 rabbit anti-mouse polyclonal antibody (M2, sc-20150, Santa Cruz Biotechnology, CA, USA), respectively.

Immunohistochemical analysis for pulmonary surfactant protein (SP)-D was performed using SP-D (H-120), a rabbit polyclonal antibody raised against amino acids 1-120 of human SP-D (SP-D (H-120): sc-13980, Santa Cruz Biotechnology, CA, USA). SP-D is a collagenous calcium-dependent lectin expressed constitutively by alveolar type 2 epithelial cells [30]. Antibodies were detected using a secondary antibody labelled with peroxidase (Histofine mouse MAX PO anti-rat and anti-rabbit, Nichirei Biosciences, Tokyo, Japan) followed by the chromogen substrate diaminobenzidine (Liquid DAB, K3468, Dakocytomation, Agilent Technologies, CA, USA). For quantification, analysis was performed in 30 images of high-power fields (×400 magnification) per slide, taken with an Evolution VR Cooled Colour 13-bit digital camera (Media Cybernetics, Canada) and manually selected under a light microscope (Nikon Eclipse 400, Nikon Instruments Tokyo, Japan). The areas occupied by nucleated macrophages and cells with positive staining for the phenotype marker in each slide were then calculated and expressed as fractional area occupied by positive cells. The images were analysed using Image Pro Plus 6.0 (Media Cybernetics, Silver Spring, MD, USA) [29, 31].

### Transmission electron microscopy

Three 2 × 2 × 2 mm slices were cut from three different segments of the right lung and fixed in 2.5% glutaraldehyde and phosphate buffer, 0.1 M (pH 7.4), for electron microscopy analysis (JEOL 1010 Transmission Electron Microscope, Tokyo, Japan). On each lung electron microscopy image (20 fields/animal), the following alterations were analysed: alveolar-capillary membrane damage, type 2 epithelial cell damage, endothelial cell injury, and macrophage changes [26]. Pathologic findings were graded on a five-point, semi-quantitative, severity-based scoring system as follows: 0 = normal lung parenchyma, 1 = changes in 1–25% of examined tissue, 2 = changes in 26–50% of examined tissue, 3 = changes in 51–75% of examined tissue, and 4 = changes in 76– 100% of examined tissue of examined tissue [26].

The pathologists and technicians working on light microscopy and TEM images were blinded to group assignment.

### Enzyme-linked immunosorbent assay

Levels of keratinocyte-derived chemokine (KC, the murine analogue of interleukin [IL]-8), tumour necrosis factor (TNF)-α, transforming growth factor (TGF)-β, and IL-10 in lung tissue were evaluated by ELISA using matched antibody pairs from PeproTech and R&D Systems (Minneapolis, MN, USA), according to manufacturer instructions. Bradford’s normalization to total protein level was done.

### Statistical analyses

Sample size calculation was based on pilot studies and on previous studies in a murine model of elastase induced emphysema conducted in our laboratory [26, 27, 29, 31, 32]. A sample size of ten animals per group would provide the appropriate power (1 − β = 0.8) to identify significant (α = 0.05) differences in mean linear intercept between C and ELA groups, taking into account an effect size d = 1.97, a two-sided test, and a sample size ratio = 1 (G ^*^- Power 3.1.9.2, University of Düsseldorf, Germany).

Normality and the equality of variance were evaluated by Kolmogorov-Smirnov test with Lilliefors’ correction and Levene’s median test, respectively. Paired *t*-tests were used to compare data between 0 and 5 weeks, while Student *t*-test or the Mann–Whitney test were used to compare C *vs* ELA at 5 weeks. Two-way ANOVA followed by Tukey’s test was used to compare C and ELA animals treated with saline or ghrelin. Parametric data were expressed as mean ± SD, while non-parametric data were expressed as median (interquartile range). The significance level was set at *P* = 0.05. All tests were performed in GraphPad Prism version 6.01 (GraphPad Software, San Diego, CA).

## Results

### Effects of ghrelin on the lungs

Est,L was significantly lower in ELA-Sal compared to C-Sal animals (Table 1). Ghrelin therapy led to increased Est,L compared to ELA-Sal animals.

**Table 1:** Lung mechanics and morphometry

	C		ELA	
	Sal	Ghr	Sal	Ghr
Lung mechanics				
Est,L (cmH_2_O mL^-1^)	41.8±12.8	37.6±4.7	18.7±2.6*	23.3±4.7#
Lung morphometry				
Lm (μm)	40.2±1.6	40.8±1.1	67.4±2.2*	50.4±1.9#
Normal (%)	99.1±0.2	98.8±0.8	49.5±1.7*	84.3±1.1#
Collapse (%)	0.9±0.0	1.2±0.8	19.3±2.0*	5.9±1.0#
Hyperinflation (%)	0.0±0.0	0.0±0.0	31.2±1.6*	9.8±1.4#
Mononuclear cells (%)	32.2±1.2	32.3±1.5	47.7±0.8*	37.3±1.4#
Neutrophils (%)	1.9±0.4	1.8±0.2	7.4±0.7*	2.2±0.5#

Values are mean ± SD of 10 animals in each group. *C* control, *ELA* elastase-induced emphysema, *Sal* i.p. injection of saline, *Ghr* i.p. injection of ghrelin, *Est,L* static lung elastance; fractional area of normal alveoli, collapsed alveoli, lung hyperinflation, mononuclear cells and neutrophils. *Significantly different from C-Sal (*P*<0.05). #Significantly different from ELA-Sal (*P*<0.05)

**Fig. 2:**
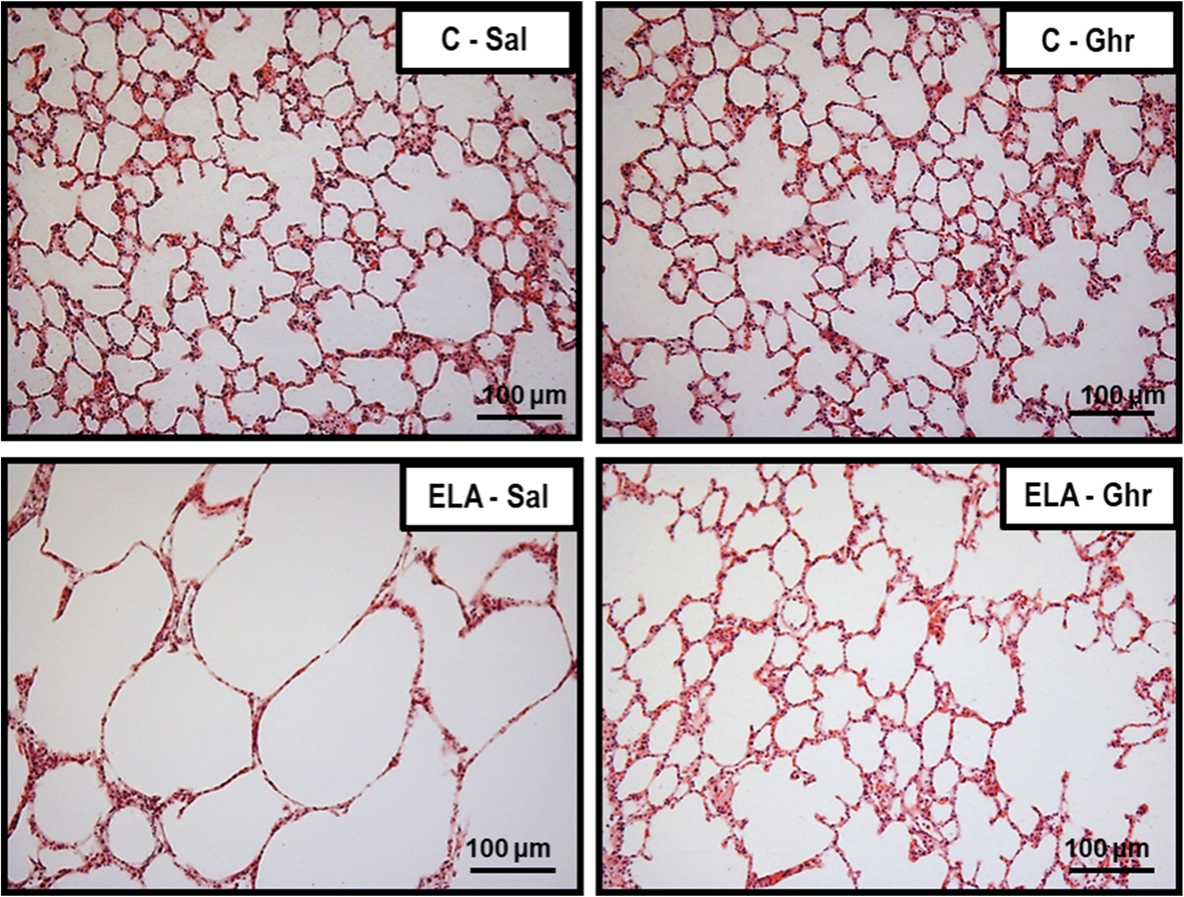
Representative photomicrographs of lung parenchyma stained with haematoxylin–eosin. C: control; ELA: elastase-induced emphysema; Sal: i.p. injection of saline; Ghr: i.p. injection of ghrelin. Original magnification ×200. Bars = 100 μm

ELA-SAL animals exhibited higher Lm, alveolar collapse, and hyperinflation, as well as a greater number of mononuclear cells and neutrophils in lung tissue, than C-Sal animals (Table 1 and Fig. 2). All of these parameters were reduced after ghrelin therapy.

**Fig. 3:**
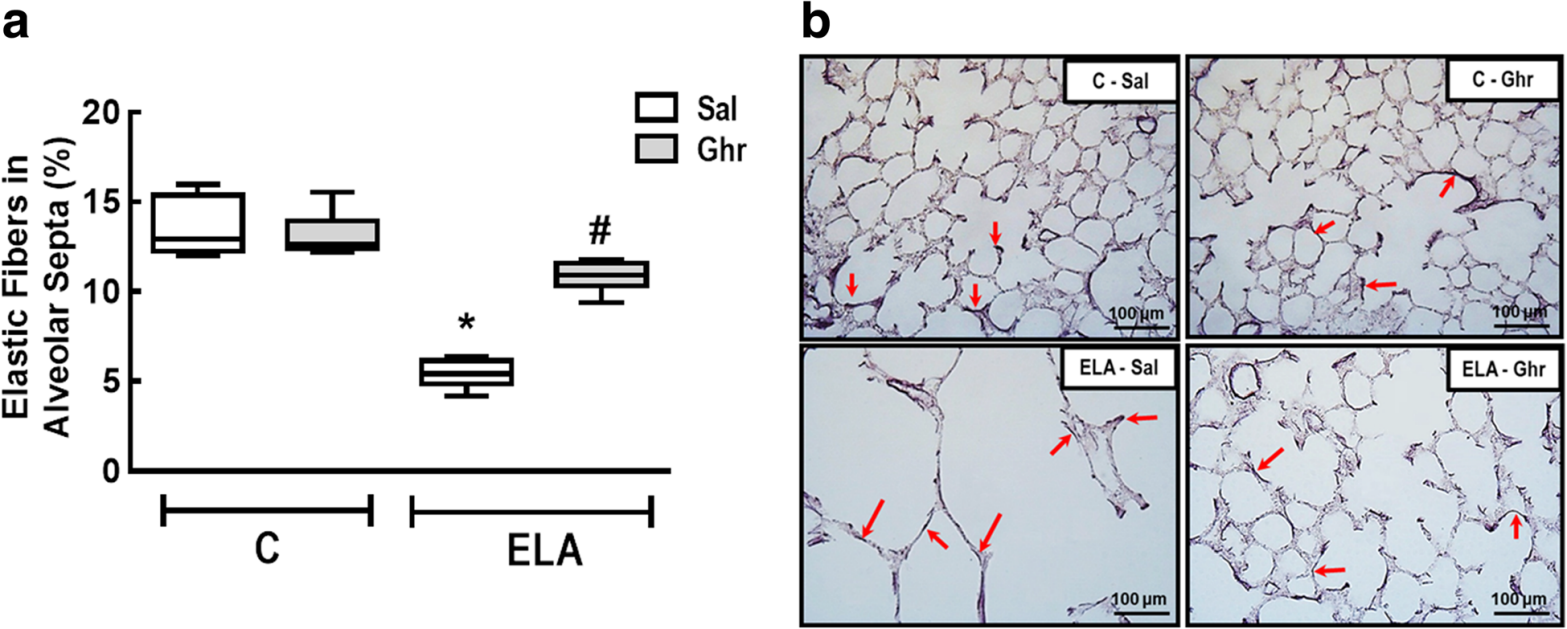
Elastic fibre content in alveolar septa (**a**) and representative photomicrographs of lung parenchyma stained with Weigert's resorcin fuchsin method with oxidation (elastic fibres) (**b**). Arrows: Elastic fibres (stained black). C: control; ELA: elastase induced emphysema; Sal: i.p. injection of saline; Ghr: i.p. injection of ghrelin. Boxes show the interquartile range (25^th^-75^th^ percentile), whiskers encompass the range (minimum–maximum), and horizontal lines represent the median in 10 animals/group. * *vs*. C-Sal. # *vs*. ELA-Sal

**Fig. 4:**
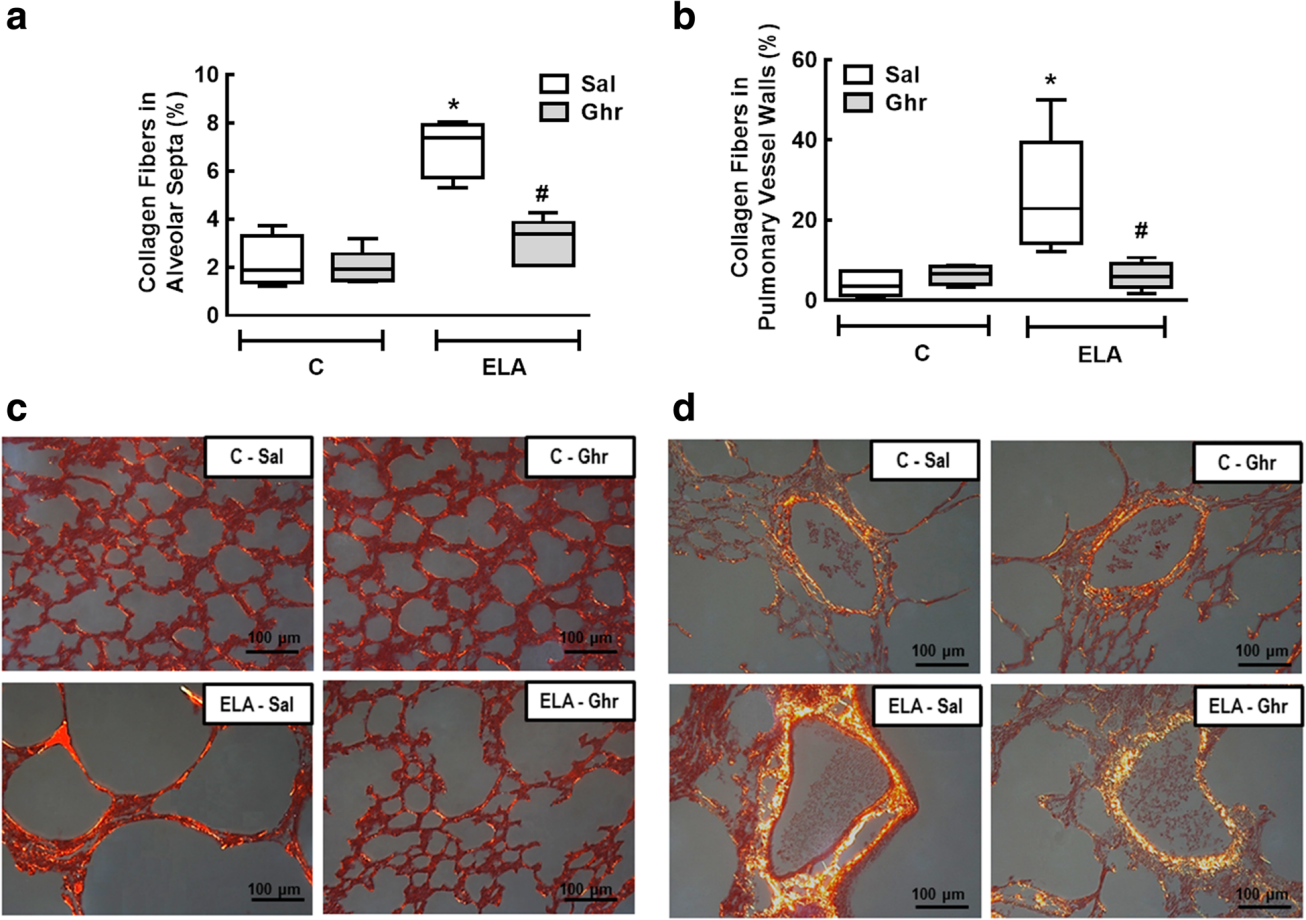
Collagen fibre content in alveolar septa (**a**) and pulmonary vessel wall (**b**). Representative photomicrographs of alveolar septa (**c)** and pulmonary vessels (**d**), stained with the Picrosirius-polarization method. Note the enlargement of alveolar space in the ELA-Sal group. C: control; ELA: elastase-induced emphysema; Sal: i.p. injection of saline; Ghr: i.p. injection of ghrelin. Boxes show the interquartile range (25^th^–75^th^ percentile), whiskers encompass the range (minimum–maximum), and horizontal lines represent the median in 10 animals/group. * *vs*. C-Sal. # *vs*. ELA-Sal

The amount of elastic fibre was reduced in ELA-Sal compared to C-Sal. Ghrelin administration increased elastic fibre content in alveolar septa (Fig. 3). Collagen fibre content in alveolar septa and pulmonary vessel walls was higher in ELA-Sal compared to C-Sal, while ghrelin minimized fibrogenesis by reducing collagen fibre deposition in both compartments (Fig. 4).

**Fig. 5:**
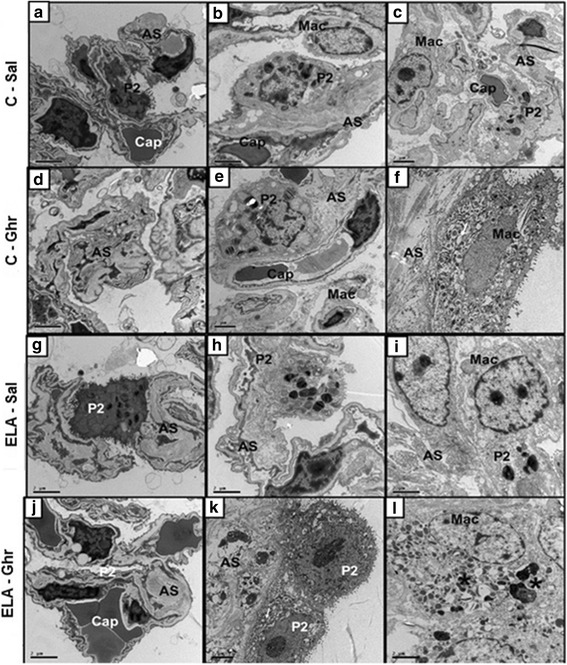
Transmission electron microscopy of lung parenchyma in control (C) animals treated with i.p. saline (Sal, **a**, **b**, and **c**) or ghrelin (Ghr, **d**, **e**, and **f**), as well as elastase-induced emphysema (ELA) mice treated with i.p. saline (**g**, **h**, and **i**) or ghrelin (**j**, **k**, and **l**). Note normal alveolar epithelium (type 2 epithelial cell, P2) and intact alveolar septa (AS) and capillary membrane (Cap) in C-Sal (**a**-**c)** and C-Ghr (**d**-**f**). Activated macrophages (Mac*) with lysosomes and glycogen accumulation can be visualized in the alveolar space in ELA-Sal (**i**). ELA-Sal animals show rupture of alveolar septa with loss of capillaries and increased collagen fibre content (in AS) (**g** and **h**). After ghrelin therapy, there is visible repair of the capillary (**j**), proliferation of type 2 epithelial cells (P2) **(k)**, suggesting epithelial repair, as well as activated macrophages with lysosomes and glycogen accumulation (**l**)

**Fig. 6:**
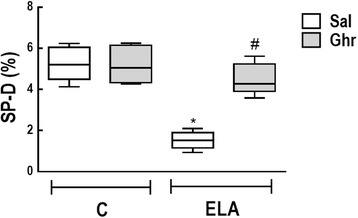
Immunohistochemistry for surfactant protein (SP)-D. Boxes show the interquartile range (25^th^–75^th^ percentile), whiskers encompass the range (minimum–maximum), and horizontal lines represent the median in 10 animals/group. * *vs*. C-Sal. # *vs*. ELA-Sal

Ultrastructural analysis of lung parenchyma in ELA-Sal demonstrated rupture of alveolar septa with loss of capillaries, epithelial and endothelial cell damage, elastolysis, and increased collagen fibre and macrophage counts. Ghrelin led to proliferation of type 2 epithelial cells (Fig. 5), reduced endothelial cell damage and collagen fibre content, and increased the number of activated macrophages (Table 2, Fig. 5). Additionally, ghrelin therapy increased the fraction area of SP-D when compared to ELA-Sal. ELA-Sal animals exhibited a reduced fraction area of SP-D compared to C-Sal animals, probably due to a reduction in SP-D protein reserve in type 2 epithelial cells secondary to emphysema (Fig. 6).

**Table 2:** Semi-quantitative analysis of electron microscopy

	C		ELA	
	Sal	Ghr	Sal	Ghr
Type 2 epithelial cell damage	0 [0-1]	1 [0-1]	3 [2.5-3.5]*	2 [2-3]
Endothelial cell damage	1 [0-1]	0 [0-0.5]	3 [3-3.5]*	2 [1.5-2.5]#
Collagen fibre	0 [0-0.5]	0 [0-0.5]	3 [2.5-3.5]*	2 [1-2]#
Activated macrophage	0 [0-1]	1 [0-1]	2 [1.5-2]	3 [2.5-3.5]*#

Values are median [interquartile range] of 5 animals per group. *C* control, *ELA* elastase-induced emphysema, *Sal* i.p. injection of saline, *Ghr* i.p. injection of ghrelin. Pathologic findings were graded on a five-point, semi-quantitative, severity-based scoring system, where 0 = normal lung parenchyma, 1 = changes in 1–25% of examined tissue, 2 = 26–50% of examined tissue, 3 = 51–75% of examined tissue, and 4 = 76–100% of examined tissue. * *vs*. C-Sal. # *vs*. ELA-Sal

**Fig. 7:**
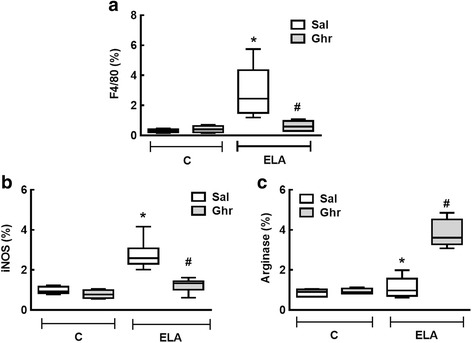
Immunohistochemistry for F4/80 (total macrophages (**a**), iNOS (M1 macrophages (**b**) and arginase-1 (M2 macrophages (**c**). C: control; ELA: elastase-induced emphysema; Sal: i.p. injection of saline; Ghr: i.p. injection of ghrelin. Boxes show the interquartile range (25^th^–75^th^ percentile), whiskers encompass the range (minimum–maximum), and horizontal lines represent the median in 10 animals/group. * *vs*. C-Sal. # *vs*. ELA-Sal

Alveolar macrophages can be activated by a variety of extracellular signals to polarize into the M1 phenotype, associated with antimicrobial response and inflammation [33], or the M2 phenotype, associated with wound healing [34] and resolution of inflammation [35]. Total macrophages (F4/80 positive cells), M1 (iNOS-positive cells) and M2 (arginase 1-positive cells) subpopulations were quantified in this study. Percentages of total, M1, and M2 macrophages were higher in ELA-Sal than C-Sal. Ghrelin administration reduced both total and M1 macrophage counts, but increased M2 macrophage counts (Fig. 7).

**Fig. 8:**
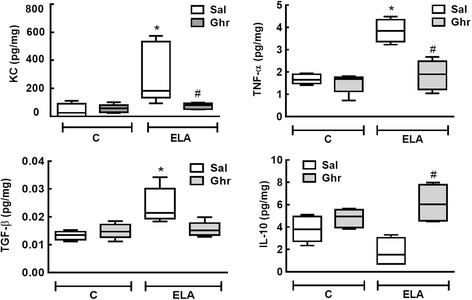
Levels of keratinocyte-derived chemokine (KC, a mouse analogue of interleukin-8), tumour necrosis factor (TNF)-α, transforming growth factor (TGF)-β, and interleukin-10. Levels in lung tissue corrected by Bradford’s method. The non-parametric Mann–Whitney test was used to evaluate between-group differences. Boxes show the interquartile range (25^th^-75^th^ percentile), whiskers encompass the range (minimum–maximum), and horizontal lines represent the median in 10 animals/group. * *vs*. C-Sal. # *vs*. ELA-Sal

KC, TNF-α, and TGF-β levels were higher in ELA-Sal compared to C-Sal animals. Compared to ELA-Sal, ghrelin treatment reduced KC, TNF-α, and TGF-β levels, but increased IL-10 levels (Fig. 8)

### Effects of ghrelin on cardiac function

**Fig. 9:**
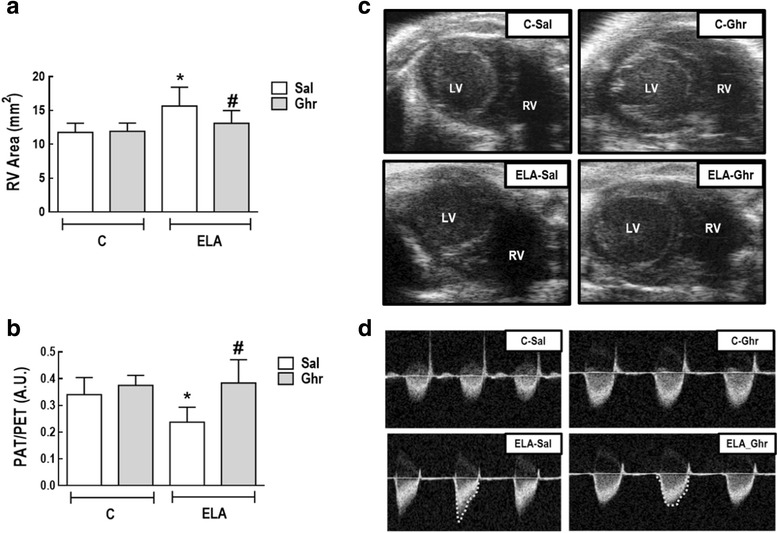
Right ventricular (RV) end-diastolic area (**a**) and pulmonary artery acceleration time/pulmonary artery ejection time (PAT/PET) ratio (**b**). Representative images of RV area on short-axis B-dimensional views of both ventricles (**c**). LV: left ventricle. Representative images of pulmonary blood flow (**d**). A.U.: arbitrary units; C: control; ELA: elastase-induced emphysema; Sal: i.p. injection of saline; Ghr: i.p. injection of ghrelin. Bars are means ± SD of 10 animals per group. * *vs.* C-Sal. # vs ELA-Sal

Cardiac function was evaluated using echocardiography. At 5 weeks, RV area increased while PAT/PET decreased in ELA in comparison to C group (Additional file 1: Table S1). At 9 weeks, RV area had increased further and PAT/PET was lower in ELA-Sal compared to C-Sal animals. Ghrelin treatment reduced RV and increased PAT/PET compared to ELA-Sal (Fig. 9). LVSV was lower in ELA-Sal compared to C-Sal animals (38.75 ± 8.99 μL and 47.06 ± 9.44 μL, respectively; P=0.016). Ghrelin had no significant beneficial effect on LVSV in either the ELA or C groups (42.79 ± 6.95 μL and 40.76 ± 8.61 μL, respectively).

### Effects of ghrelin on body composition

At 5 weeks, lean mass had decreased and fat mass had increased in the ELA group (Additional file 2: Table S2), but total mass did not differ between the ELA and C groups.

At 9 weeks, ELA-Sal animals presented less total mass and lean mass than C-Sal animals. Ghrelin treatment promoted an increase in total mass and lean mass compared to ELA-Sal. No significant differences in body composition were observed between C-Sal and C-Ghr animals (Table 3).

**Table 3:** Body composition

	C		ELA	
	Sal	Ghr	Sal	Ghr
Total mass (g)	20.1±0.9	19.8±1.1	13.2±0.8*	18.8±1.8#
Fat mass (g)	5.4±2.0	6.0±2.9	5.0±1.0	7.4±1.0
Lean mass (g)	11.6±2.3	13.2±2.9	8.2±1.1*	11.0±1.9#

Values are means (±SD) of 10 animals in each group. *C* control, *ELA* elastase-induced emphysema, *Sal* i.p. injection of saline, *Ghr* i.p. injection of ghrelin. * *vs.* C-Sal. # *vs.* ELA-Sal

## Discussion

In the experimental model of elastase-induced emphysema used in this study, ghrelin therapy had several effects on the lungs. It decreased inflammation, altered the macrophage milieu (increasing M2 while reducing M1 macrophage counts), decreased collagen fibre content in the alveolar septa and pulmonary vessel walls, increased elastic fibre content in the alveolar septa, reduced alveolar hyperinflation and collapse, and ultimately improved lung mechanics. From an extrapulmonary standpoint, ghrelin mitigated the cardiovascular impairment observed in emphysema animals, and increased total and lean body mass. To the best of our knowledge, this was the first study to analyse the potential therapeutic effects of ghrelin on lung and cardiac function as well as pulmonary inflammation and remodelling in experimental emphysema.

### Elastase-induced emphysema model

The use of porcine pancreatic elastase (PPE) to induce emphysema experimentally is more advantageous than cigarette smoke exposure, as PPE is inexpensive and able to induce greater and more widespread lung damage [36–38]. Both models may result in cachexia [39] and pulmonary hypertension [40], but the lung damage induced by elastase persists for longer after induction in contrast to cigarette smoke [41]. In addition to lung structural damage induced by PPE, a persistent lung inflammatory process [42] with elastolysis and fibrogenesis has been observed [32]. The specific elastase-induced emphysema protocol used in the present study has been shown to produce loss of lean and total body mass, likely suggesting cachexia [43].

### Ghrelin in emphysema

The abnormalities of emphysema alterations are not confined to the lungs, but also involve extrapulmonary organs [44, 45]. Adipose tissue and lean mass express ghrelin receptors to maintain energy balance, according to respective plasma level [46]. By acting on adipose tissue cells, ghrelin reduces the release of leptin, which has pro-inflammatory effects, while increasing adiponectin levels, leading to anti-inflammatory effects [47]. In this line, it has been shown that ghrelin treatment decreases production of the pro-inflammatory cytokines IL-1, IL-6, and tumour necrosis factor (TNF)-α [47], as well as neutrophil density in sputum from COPD patients [48], and increases levels of anti-inflammatory cytokines, such as IL-10 [10]. The dosage of ghrelin used herein (200 μg kg^-1^ per day) was chosen based on pooled data from previous experimental [49, 50] and clinical [15, 48] studies.

### Pulmonary effects of ghrelin treatment in emphysema

Lung inflammation is one of the hallmarks of emphysema [4, 42]. Contributing to this feature, alveolar macrophages can be activated by several extracellular signals to polarize into the M1 or M2 phenotypes. At the early stages of inflammation, macrophages are functionally distinct from those at later stages. Early-phase macrophages are predominantly M1-biased cells and contribute to extracellular matrix deposition and fibrosis, likely producing pro-fibrotic cytokines. During the late resolution phase, macrophages tend to be alternatively activated, remodelling-competent, M2-biased macrophages. Although this is not fully understood, the resolution of scarring and fibrosis appears to be – unsurprisingly – the responsibility of total macrophages and, in particular, M2 macrophages [51].

The enhanced M1 polarization observed after elastase instillations in our experiment was in agreement with previous studies [52, 53]. KC is one of the major cytokines involved in the pathophysiology of emphysema, as it is produced by macrophages and has the function of attracting neutrophils to the lungs [54]. Additionally, ghrelin is expressed in monocytes and, via its receptor, inhibits the expression of pro-inflammatory cytokines [55]. In our study, the reduction in M1 promoted by ghrelin treatment was accompanied by a significant reduction in levels of the pro-inflammatory cytokines TGF-β and TNF-α and neutrophil counts in lung tissue. Ghrelin also increased the M2 subpopulation in emphysema animals and increased IL-10 levels, thus contributing to a reduction in the inflammatory process.

Emphysema is characterized by changes in the organization and composition of the extracellular matrix [3, 56, 57]. After elastase administration, elastolysis and fibrosis in both alveolar septa and pulmonary vessel walls were detected, leading to loss of elastic recoil and decreased elastance [58, 59]. In liver fibrosis, ghrelin has been found to inhibit extracellular matrix formation by maintaining the balance between matrix metalloproteinases (MMP2) and tissue inhibitor of matrix metalloproteinases (TIMP1) [60]. In addition, by promoting activation of the M2 macrophage subpopulation, ghrelin may foster wound healing [61] and, likely, elastogenesis. Moreover, ghrelin therapy reduced alveolar hyperinflation and collapse in our sample, leading to alveolar stabilization and improvement in lung mechanics.

Increased total collagen expression in the airway and parenchymal compartment has been reported in mild-to-moderate and severe COPD [62]. In the present study, total collagen was significantly reduced in the lung parenchyma of ELA animals treated with ghrelin.

### Extrapulmonary effects of ghrelin treatment in emphysema

In the clinical setting, the effects of ghrelin on cardiorespiratory function are controversial; conflicting findings may be attributable to differences in ghrelin dose, small sample sizes, or both [19]. In the present study, ghrelin treatment was associated with a reduction in pulmonary arterial hypertension, as detected by PAT/PET ratio and RV area as evaluated by echocardiography. Since right ventricular function is directly related to lung vessel density, we may speculate that ghrelin acted not only through vasodilation [14, 15], but also by decreasing collagen fibre deposition on lung vessel walls [63]. Reduction of right ventricular area may also contribute to decompression of the left ventricular area and, consequently, indirectly lead to an increase in LVSV (ventricular interdependence). However, no statistically significant changes in LVSV were observed in our study, which may be attributed to the timing of analysis.

Although we did not observe differences in body weight among emphysema animals, we did observe differences in body composition as analysed by DEXA. Ghrelin treatment increased lean mass and total body mass in the ELA group. Thus, in the elastase-induced emphysema model used herein, ghrelin seems to result in beneficial effects on body composition and cardiovascular function, two important factors associated with worse prognosis in COPD.

### Limitations

This study has some limitations. First, no experimental model of emphysema reproduces all features of the human disease. However, the present model of emphysema induced by multiple elastase instillations was associated with cardiorespiratory functional changes and loss of total and lean mass, and may provide an efficient tool to better understand the effects of ghrelin when the disease is already established, with potential translation into clinical practice. Second, our results cannot be extended to other emphysema models with different triggers or different degrees of severity. Third, we evaluated the effects of ghrelin at only one time point (9 weeks). Further studies are required to analyse the time course of ghrelin therapy in experimental elastase-induced emphysema.

## Conclusions

In the emphysema model used in this study, ghrelin treatment led to beneficial pulmonary and extrapulmonary effects, mitigating lung damage, improving cardiovascular function, and reverting body mass losses. Although the precise mechanism behind this effect remains unknown, supplemental treatment with ghrelin may represent an interesting alternative for treatment of emphysema.

## Additional files

Additional file 1: **Table S1.** Echocardiographic parameters in the randomised groups. (DOCX 13 kb)

Additional file 2: **Table S2.** Body composition in the randomised group. (DOCX 13 kb)

## Abbreviations

ANOVA: analysis of variance
C: control
COPD: chronic obstructive pulmonary disease
DEXA: dual-energy X-ray absorptiometry
ELA: emphysema
ELISA: enzyme-linked immunosorbent assay
Est,L: static lung elastance
FiO2: fraction of inspired oxygen
Ghr: ghrelin
i.p.: intraperitoneally
IL: interleukin
KC: keratinocyte-derived chemokine
LV: left ventricle
LVSV: left ventricular stroke volume
PAT: pulmonary artery ejection time
PEEP: positive end-expiratory pressure
Pel: lung elastic recoil pressure
PET: pulmonary ejection time
PL: transpulmonary pressure
PPE: pancreatic porcine elastase
Ptr: tracheal pressure
RV: right ventricle
Sal: saline
TEM: transmission electron microscopy
V_T_: tidal volume
