# Supplementary material for: Ghrelin therapy improves lung and cardiovascular function in experimental emphysema
Source: Respir Res. 2017 Nov 3;18:185. doi: 10.1186/s12931-017-0668-9 (PMC5670513; doi:10.1186/s12931-017-0668-9)
Supplement: Supplementary file 1 — Table S1. Echocardiographic parameters in the randomised groups. (DOCX 13 kb) [file 12931_2017_668_MOESM1_ESM.docx]

**Table S1**

Echocardiographic parameters in the randomised groups

| Parameter | Group | Week | | P-value |
| --- | --- | --- | --- | --- |
|  |  | 0 | 5 | (C *vs*. ELA at 5 weeks) |
| RV area (mm^2^) | C | 10.5±0.8 | 10.5±1.2 | 0.086 |
|  | ELA | 10.2±0.9 | 12.1±1.1 | 0.002 |
| PAT/PET (A.U.) | C | 0.38±0.1 | 0.35±0.0 | 0.488 |
|  | ELA | 0.45±0.0 | 0.31±0.1 | 0.019 |
| LVSV (mL) | C | 34.9±6.1 | 31.9±8.6 | 0.089 |
|  | ELA | 33.7±7.1 | 31.2±8.2 | 0.168 |

Values are means (±SD) of 10 animals in each group. C: mice treated with saline. ELA: mice treated with elastase. RV: right ventricle; PAT/PET: pulmonary artery acceleration time/pulmonary artery ejection time; LVSV: left ventricular stroke volume. The Student *t*-test was used to compare data between C and ELA groups at 5 weeks. No significant differences were observed between C and ELA groups at week 0.
