# Supplementary material for: Ghrelin therapy improves lung and cardiovascular function in experimental emphysema
Source: Respir Res. 2017 Nov 3;18:185. doi: 10.1186/s12931-017-0668-9 (PMC5670513; doi:10.1186/s12931-017-0668-9)
Supplement: Supplementary file 2 — Table S2. Body composition in the randomised group. (DOCX 13 kb) [file 12931_2017_668_MOESM2_ESM.docx]

**Table S2**

Body composition in the randomised groups

| Parameter | Group | Week | | P-value |
| --- | --- | --- | --- | --- |
|  |  | 0 | 5 | (C *vs*. ELA at 5 weeks) |
| Total mass (g) | C | 18.7±0.7 | 21.0±1.0 | 0.001 |
|  | ELA | 17.6±1.1 | 20.3±1.3 | 0.002 |
| Fat mass (g) | C | 7.0±1.8 | 9.1±2.5 | 0.041 |
|  | ELA | 6.7±1.9 | 10.9±2.2 | 0.001 |
| Lean mass (g) | C | 11.2±2.1 | 10.8±2.0 | 0.162 |
|  | ELA | 10.6±2.5 | 8.2±2.2 | 0.009 |

Values are means (±SD) of 10 animals in each group. C: mice treated with saline. ELA: mice treated with elastase. A paired *t*-test was used to compare data between 0 and 5 weeks, while the Student *t*-test was used to compare data between C and ELA groups at 5 weeks. No significant differences were observed between C and ELA groups at week 0.
